# Improving allied health professionals’ research implementation behaviours for children with cerebral palsy: protocol for a before-after study

**DOI:** 10.1186/s13012-014-0202-0

**Published:** 2015-02-06

**Authors:** Christine Imms, Iona Novak, Claire Kerr, Nora Shields, Melinda Randall, Adrienne Harvey, H Kerr Graham, Dinah Reddihough

**Affiliations:** Australian Catholic University, 17 Young Street, Fitzroy, Melbourne, 3065 Australia; Cerebral Palsy Alliance, Frenchs Forest, PO Box 6427, NSW 2086 Sydney, Australia; School of Medicine, University of Notre Dame Australia, Broadway, PO Box 944, 2007 Sydney, Australia; La Trobe University, Kingsbury Drive, Bundoora, 3086 Victoria Australia; Northern Health, 1231 Plenty Road, Bundoora, 3083 Victoria Australia; Victorian Paediatric Rehabilitation Service, Murdoch Childrens Research Institute, 50 Flemington Road, Parkville, 3052 Australia; Hugh Williamson Gait Laboratory, Royal Children’s Hospital, Parkville, Australia; University of Melbourne, Parkville, Australia; Murdoch Children’s Research Institute, Parkville, 3052 Australia

**Keywords:** Cerebral palsy, Knowledge translation, Evidence-based practice, Surveillance, Assessment, Child

## Abstract

**Background:**

Cerebral palsy is a permanent disorder of posture and movement caused by disturbances in the developing brain. It affects approximately 1 in every 500 children in developed countries and is the most common form of childhood physical disability. People with cerebral palsy may also have problems with speech, vision and hearing, intellectual difficulties and epilepsy. Health and therapy services are frequently required throughout life, and this care should be effective and evidence informed; however, accessing and adopting new research findings into day-to-day clinical practice is often delayed.

**Methods/Design:**

This 3-year study employs a before and after design to evaluate if a multi-strategy intervention can improve research implementation among allied health professionals (AHPs) who work with children and young people with cerebral palsy and to establish if children’s health outcomes can be improved by routine clinical assessment. The intervention comprises (1) knowledge brokering with AHPs, (2) access to an online research evidence library, (3) provision of negotiated evidence-based training and education, and (4) routine use of evidence-based measures with children and young people aged 3–18 years with cerebral palsy. The study is being implemented in four organisations, with a fifth organisation acting as a comparison site, across four Australian states. Effectiveness will be assessed using questionnaires completed by AHPs at baseline, 6, 12 and 24 months, and by monitoring the extent of use of evidence-based measures. Children’s health outcomes will be evaluated by longitudinal analyses.

**Discussion:**

Government, policy makers and service providers all seek evidence-based information to support decision-making about how to distribute scarce resources, and families are seeking information to support intervention choices. This study will provide knowledge about what constitutes an efficient, evidence-informed service and which allied health interventions are implemented for children with cerebral palsy.

**Trial registration:**

Trial is not a controlled healthcare intervention and is not registered.

## Background

Children with cerebral palsy have a ‘permanent disorder of the development of movement and posture, causing activity limitations that are attributed to non-progressive disturbances that occurred in the developing fetal or infant brain. The motor disorders of cerebral palsy are often accompanied by disturbances of sensation, perception, cognition, communication and behaviour, by epilepsy and by secondary musculoskeletal problems’ [1]. Cerebral palsy is the most common cause of physical disability in childhood, with a prevalence of approximately 2.0 per thousand live births [2]. In 2007, it was estimated that 33,797 people with cerebral palsy were living in Australia [3]. Recent research has found that children with cerebral palsy may receive ineffective interventions despite numerous systematic reviews that report on the efficacy of specific therapeutic interventions in this population [4,5]. This finding is consistent with other research that shows between 10% and 40% of all patients do not receive treatments that have demonstrated high levels of effectiveness, and more than 20% of patients receive ineffective or harmful treatments [6]. It is therefore apparent that a research-practice gap exists in healthcare and specifically in relation to allied health professional (AHP) management of children with cerebral palsy.

One pertinent example of the consequences of a research-practice gap in the management of children and young people with cerebral palsy is the development of secondary musculoskeletal deformities that are a common feature of the condition. These deformities can include hip dislocation, which may result in pain, contractures and problems with functional activities such as walking, fractures, skin ulceration and difficulty with perineal care, pelvic obliquity and scoliosis [7]. In Australia, the overall incidence of hip displacement, which may progress to dislocation, is 35% in children with cerebral palsy [8]. While Australian hip surveillance guidelines were initially published in 2008 [9], and revised more recently [10], there is currently no knowledge as to whether there has been uniform adoption of these guidelines and no consistent mechanism for assisting AHPs to implement this evidence-based management approach. There is evidence that hip displacement can be detected for children who are enrolled in a surveillance programme [11-13]. Once detected, an opportunity exists for further active management, including consideration for surgery. Earlier detection of progression of hip displacement has been shown to lead to successful outcomes following surgery [7] and the abolition of salvage surgery [7,13]. Severe orthopaedic deformities among children with cerebral palsy have been almost eliminated in Sweden as a result of the implementation of a consistent surveillance programme and responsive surgery [13-16]. Successful surveillance provides an opportunity for more effective management of the musculoskeletal deformities associated with cerebral palsy and can reduce the significant consequences of ‘late referral’ such as the cost to children and families in receiving ineffective assessment or treatments and the cost to the tax payer of delivering an inadequate or ineffective service.

Knowledge translation is required to close the ‘research-practice gap’ [17] for children with cerebral palsy. It is defined as ‘the exchange, synthesis and ethically sound application of knowledge…to accelerate the capture of the benefits of research for [people] through improved health, more effective services and products, and a strengthened health care service’ [18]. Research utilisation and implementation describe the processes and actions by which specific research is implemented or executed within practice [18].

A number of barriers to implementing research evidence in clinical practice have been identified [19,20] which relate to the individual health practitioners, their workplace, their educational experiences and their patients [21]. In particular Haines et al. [19] and Sitzia [22] identified that:

1. Individual practitioner barriers included obsolete knowledge, influence of opinion leaders who may go against research evidence and personal beliefs and attitudes, including a perception among many health professionals that they lack the authority to change clinical practice [20].
2. Workplace barriers included a lack of financial and human resources, policies that did not promote cost-effective interventions or advocated unproven interventions, lack of access to evidence-based data, time limitations and a lack of disease registers.
3. Educational system barriers included a failure of curricula to reflect current research evidence, inappropriate continuing professional development or education and a lack of incentives to participate in effective educational activities.
4. Patient barriers included demands for care that is ineffective and perceptions or cultural beliefs about appropriate care.

Educational strategies have been traditionally used with the aim of bridging the research-practice gap. Such strategies include provision of educational materials, promoting attendance at conferences and professional development courses, establishing interactive small-group meetings within departments, use of opinion leaders and feedback on performance [6,19,20]. In spite of the diverse range of practical and applied strategies detailed above, systematic reviews have consistently shown that these approaches induce only a 6% improvement in health professionals’ evidence-based practice knowledge and have little influence on how AHPs actually practice [19,23,24]. Education or single strategies alone have been thought to be insufficient for changing the behaviour of health professionals in implementing research evidence [25-27], and tailored, complex interventions that seek to redress workplace barriers to research implementation have been recommended [19,28-30]. Other recommended strategies include (a) information technology support through the introduction of computers into practice to enable access to online evidence summaries and computerised decision supports to link individual health information with health knowledge to aid clinical decision-making [6,19,20,31] and (b) workplace alterations such as encouraging multi-professional collaboration, fostering a research culture and quality management or improvement strategies [19,20,31]. More recently, a systematic review of systematic reviews, published after the initiation of this trial, concluded that the evidence for multifaceted interventions, compared to single-component interventions, in changing healthcare professionals’ behaviours was not compelling [32]. No statistical evidence of a relationship between the number of interventions provided and the effect size was observed; however, the review commented that methods of categorising intervention elements were not transparent, and so delineation between ‘single’ versus multifaceted interventions may be difficult [32].

A single-blind clustered randomised controlled trial (RCT) has investigated the effect of a tailored complex intervention on the research implementation of AHPs that work with children with cerebral palsy in a single organisation [33]. The tailored multifaceted intervention comprised (a) redressing workplace barriers in partnership with knowledge brokers and (b) providing a customised intranet resource consisting of an e-evidence library of synthesised and critiqued cerebral palsy research, a suite of decision-making intervention algorithms based on best available evidence and the latest research on cerebral palsy prognosis and assessment. The study demonstrated statistically significant differences between the intervention and control groups in evidence-based practice knowledge. Improvements in self- and peer-rated evidence-based practice behaviours were found for the intervention group relative to the control group; however, the difference was not statistically significant after adjusting for the clustering effect [33]. Campbell and colleagues [31] demonstrated that a specific package of strategies may improve knowledge and behaviour in a particular organisation; however, it is not known whether the strategies can effectively be applied across varying organisations within the health and disability sector.

This paper describes the study protocol for a partnership project funded by the National Health and Medical Research Council of Australia. The ethically approved project title is *Cerebral palsy check up: providing the best service at the best time.* This paper describes the rationale and methodology for a ‘real-world’ implementation study that aims to improve evidence-based practice behaviours of AHPs that work with children with cerebral palsy via the application of a tailored multi-strategy knowledge translation intervention. The intervention will be applied pragmatically in ‘real-world’ settings in different disability service organisations in Australia.

### Conceptual framework

This project is guided by the diffusion, dissemination and implementation of innovation conceptual framework described by Greenhalgh et al. [34]. This model describes characteristics of successful innovations and diffusion and dissemination methods for implementing new ways of practicing. Innovations are defined as a ‘novel set of behaviours, routines and ways of working that are directed at improving health outcomes, administrative efficiency, cost effectiveness, users’ experience and that are implemented by planned and coordinated actions’ ([34], p. 582). When describing the spread of innovation in service organisations, Greenhalgh et al. differentiated three conceptual and theoretical bases, ‘let it happen’, ‘help it happen’ and ‘make it happen’ and defined their key features ([34], p. 593). Our study will address the ‘help it happen’ concept by negotiating, influencing and enabling the use of evidence-based practice by AHPs that work with children with cerebral palsy.

### Overall purpose and hypotheses

This study aims to *improve allied health professional research implementation behaviours* using a tailored multi-strategy intervention package [33] and to reduce the research-practice gap in multiple organisations that provide services to children with cerebral palsy.

Our specific research questions include

1. Does the tailored multi-strategy intervention change AHP behaviours via greater use of evidence-based outcome measures and/or interventions, and improved AHP knowledge of evidence-based interventions and outcome measures for children with cerebral palsy?
  - How does AHP knowledge of evidence-based interventions and outcome measures for children with cerebral palsy in the four organisations in which the intervention is introduced compare to the organisation in which the practices are mandated?
2. What is the relationship between the numbers and/or type of barriers to knowledge translation and the level of uptake of routine use of outcome measures in different healthcare organisations?
  - How do barriers in the four organisations in which the intervention is introduced compare to the organisation in which the practices are mandated?
3. How does the size and scope of the organisation impact the implementation of the multi-strategy approach?
4. Do time trends show a reduction in rates of adverse outcomes, including severe scoliosis, hip dislocation (MP >100%) and progressive limb contractures over the study period, for children with cerebral palsy whose parents consent to be a part of this project? If so, are these trends comparable to Swedish cerebral palsy follow-up outcomes?

## Methods/design

### Research design

This study will employ a before and after design [35] in four Australian non-government organisations that provide allied health services to children with cerebral palsy, with a fifth organisation acting as a comparison site. Included organisations are Yooralla and Kids Plus in Victoria, St Giles in Tasmania, Novita Children’s Services in South Australia and Cerebral Palsy Alliance in New South Wales. Cerebral Palsy Alliance will act as a comparison site as the tailored intervention is mandated in that organisation and embedded in routine practice following a randomised controlled trial [33]. By mandating these practices, the Cerebral Palsy Alliance is deemed to be operating according to the ‘make it happen’ concept ([34], p. 593). Having a comparison site in which the tailored intervention is in routine use affords the opportunity to examine the process of diffusion of innovation and describe features that may account for the success or failure of the tailored intervention in different organisations, as recommended by Greenhalgh et al. ([34], p. 615–6), and articulated in research questions two and three above. Measures of clinician practices at each site will be collected at baseline (prior to implementation of the intervention) and at 6, 12 and 24 months following implementation. The total study duration is 3 years.

### Participants and recruitment

1. AHPs: All physiotherapists, occupational therapists and speech pathologists working, or having the potential to work with children with cerebral palsy in participating service providers, will be eligible to participate. Organisation-specific recruitment strategies will be implemented including advertising posters, newsletter commentaries, email contact and face to face sessions for staff and families. Written informed consent will be sought from AHPs; consent to participate is voluntary, and there will be no adverse consequences for any eligible participant who chooses not to take part.
2. Children and families: Children aged 3–18 years with a diagnosis of cerebral palsy will also be recruited to the study. All families whose children with cerebral palsy receive services at participating organisations will be provided with detailed information about the study. Consent to collect longitudinal child-specific data to monitor health service inputs and outcomes related to the project will be sought. Again, consent to participate will be voluntary, and there will be no adverse consequences for any eligible participant who chooses not to take part.

### Ethics

Ethical approval for this study has been granted from the Australian Catholic University Research Ethics Committee (Reference: 2013-309 V), the Department of Education and Early Childhood Development (Reference: 2013_001962) and the Cerebral Palsy Alliance Research Ethics Committee (Reference: 2013-04-02). Approval documents from the Australian Catholic University Research Ethics Committee were lodged and accepted by each partner organisation’s ethics committee or chief executive officer.

### Intervention

The tailored multi-strategy intervention aims to address known barriers to evidence use and will consist of:

1. Identification of ‘*Knowledge Brokers*’ (expert research translators) based at each organisation who will aim to redress workplace barriers unique to each site. Each organisation will nominate one or more knowledge brokers for their site who has demonstrated leadership skills, excellent communication skills, peer respect and a specific interest in evidence-based practice. The knowledge brokers will work with the project team, the organisation’s management and the AHPs to act as agents in the process of translating and contextualising evidence for users within the organisation. This will involve taking part in training and cross-organisational knowledge broker support groups to develop the knowledge brokering role, identifying facilitators and ameliorating context-specific barriers, contributing to the delivery of education packages and providing day-to-day support to AHPs as they engage in project activities and outcomes.
2. Provision of a *customised e-evidence library*—‘*CP Decision*’ [36]—to enable AHPs rapid and real-time access to synthesised and critiqued cerebral palsy research evidence. This library employs a traffic light system corresponding to evidence levels [21] and will be updated on an ongoing basis to accommodate new evidence as it emerges. The resource also contains a suite of decision-making intervention algorithms based on best available evidence in addition to research on cerebral palsy prognosis and assessment. The purpose of providing *CP Decision* is to decrease the amount of time it takes AHPs to retrieve the highest levels of published evidence.
3. *Negotiated education and professional development days* for AHPs at partner organisations (implemented by the project investigators and the organisation-based knowledge brokers) about ‘how to’ implement or administer the current and most effective treatment and measurement options for cerebral palsy, using concrete and precise descriptions of the clinical processes involved.
4. Provision of an *electronic cerebral palsy clinical outcomes database* (CP Check-Up™, provided by Cerebral Palsy Alliance). This electronic tool will be used by AHPs to record routine valid and reliable clinical measurements of participating children and to summarise the therapeutic interventions provided to these children. Through instant generation of an electronic report, the tool will provide real-time feedback about changes in outcomes of individual children over time, guiding evidence-based clinical decision-making to support direct therapeutic intervention and timely referral to ancillary services.

### Outcome measures

*Change in AHP behaviour* (use of evidence-based outcomes and interventions, research question 1) will be determined in two ways:

(i). The Best Service Best Time Evaluation questionnaire of evidence-based practice behaviours reported by participating AHPs (adapted from Campbell et al. [33] and Aarons [37]) will be collected on four occasions: baseline, 6, 12 and 24 months. In addition to participant demographics, the questionnaire describes evidence-based practice behaviours such as client goal setting, use of research and use of measures to inform clinical decision-making.
(ii). Data extracted from the electronic cerebral palsy clinical outcomes database. This tool and associated database has been developed for the Australian context by Cerebral Palsy Alliance, the study comparison site, and is based on the Swedish cerebral palsy follow-up surveillance programme [14-16]. It is used to collate data from a range of evidence-based outcome measures and to record interventions provided. A minimum dataset within the clinical outcomes database has been defined for this project and includes elements related to the general description of cerebral palsy type, distribution, associated impairments, mobility and lower limb functions, upper limb and self-care functions, communication and swallowing abilities and nutrition.

Children aged less than 6 years will be assessed at 6 monthly intervals; children aged 6–18 years will be assessed at baseline, 6, 12 and 24 months. Data extracted from the clinical outcomes database to determine change in AHP behaviour will include proportion of eligible professionals routinely contributing data, proportion of clinical outcome measures completed per child, proportion of cerebral palsy caseload with data reported and comparisons between expected proportions in the population who might be eligible for specific management, such as hip surveillance, and the observed proportions receiving them.

*Change in AHP evidence-based practice knowledge* (research question 1) will be measured using the Evidence Based Practice and Outcome Measurement Competency quiz [33] that has been integrated into the Best Service Best Time Evaluation for the purposes of this study. This knowledge quiz includes open-ended questions developed by a panel of experts and assesses AHP’s knowledge of reliable, valid measures used in cerebral palsy and the current level of evidence for interventions used for children with cerebral palsy. This measure will be implemented at baseline, 6, 12 and 24 months.

*Impact of organisational factors on implementing the strategies* (research questions 2 and 3) will be assessed using mixed methods, including a questionnaire that evaluates AHP perception of the extent to which organisational factors act as supports or barriers to implementation of evidence-based practices (Supports and Barriers Questionnaire, adapted with permission from [38]) and qualitatively through focus groups and interviews. Focus groups will include three groups of participants, each with representatives from the participating organisations: (1) knowledge brokers, (2) clinical services managers and (3) participating AHPs. Interviews will be conducted with senior managers or chief executive officers.

*Longitudinal outcomes of children with cerebral palsy* (research question 4) will be assessed using clinical data extracted from the clinical outcomes database. Specific musculoskeletal measurements will be modelled from data collected at 6, 12 and 24 months.

Additional data relating to potential confounders for uptake of evidence-based practices will be collected including organisational characteristics such as profile of children attending the service (multiple diagnoses or only cerebral palsy), size and number of sites that an organisation operates from and professional characteristics of AHPs including discipline, years of experience and proportion with higher degrees. In addition, a record of implementation at each site, including number and timing of recruitment of knowledge brokers and staff, days and topics of professional development provided and frequency of hits (number of times accessed) on the e-library, will be kept to provide process measures for the study.

### Statistical analyses

Statistical plans addressing each specific research question will be pre-specified, to prevent bias due to post hoc analyses. Cross-sectional data will be examined and reported; however, primary analyses will focus on the longitudinal design of this study, assessing change over time in AHP evidence-based practice behaviours and in clinical outcomes for participating children.

Quantitative analyses will be carried out with multivariate statistical models such as population-averaged generalised estimating equations and subject-specific generalised linear and latent mixed models. These models allow for correlated longitudinal data structures and intraclass correlation coefficients (required due to children being clustered within AHPs and AHPs within organisations). In addition, these models can include outcomes of mixed types including counts, ordered and unordered responses, and dichotomous and metric outcomes. Potential confounders will be examined, and study sites will be treated as covariates to account for differing adoption of new practice rates. The significance of the covariates will be ascertained with 95% confidence intervals.

Qualitative focus group and interview data will be digitally recorded and transcribed, coded and analysed using a grounded theory approach to thematic analysis [39]. Themes within and between participants, and groups of participants, will be used to understand the feasibility and acceptability of the knowledge broker role and aligned evidence-based practices within the different organisations.

### Estimated sample size and power of the study

Based on organisational commitment to the project, and expected workforce changes during the course of the study, it is anticipated that 80% of the eligible AHPs will take part in the study (i.e. 192 participants from an estimated 240 eligible AHP participants across all partner organisations). Calculations indicate that this sample will be sufficient to detect a difference of 6 points on the Best Service Best Time Evaluation with >90% power.

Pre-study estimation of the number of participating children suggests a sample size of 614 may be achieved (80% of the 768 eligible children identified across all partner organisations).

A 10% arithmetic difference between baseline and subsequent child-based quality measures (e.g. range of movement) will be able to be detected with >90% power (where baseline percentage is around 50%; power will be greater where the baseline percentage is smaller or larger than 50%).

## Governance and quality

### Study oversight

Governance for the project will include a project steering committee, partner organisation local advisory groups and a data monitoring committee. *The project steering committee* will comprise an external chair, the chief investigators, one associate investigator from each partner organisation, the project manager and a consumer representative. The steering committee will hold four face-face meetings in the course of the project and meet monthly by teleconference. *Local advisory committees* will be established in each partner organisation. At a minimum, these committees will include an associate or chief investigator, who also sits on the steering committee, one knowledge broker, one AHP leader/manager from within the organisation and a consumer representative. These groups will oversee the site-specific implementation of the project, identify and ameliorate risks to implementation, ensure record keeping associated with project implementation is maintained and report monthly to the steering committee on project progress. *A data monitoring committee* will also be established, consisting of the chief investigator, project manager and two other members of the research team to monitor the implementation of the project and audit data collection processes. Day-to-day management of the project will be undertaken by a full-time project manager supported by one part-time research assistant per state.

### Data quality procedures

To ensure integrity in the management of the project across multiple organisations and to safeguard participating AHPs and patients’ rights and the security and quality of data, project-specific standard operating procedures will be developed and provided to each organisation for implementation. Standards will be developed in the following areas: procedure for consenting AHPs, procedure for consenting children and families, clinical data collection and electronic data entry.

Data will be managed and analysed centrally. Site-specific consent forms and AHP questionnaire data will be scanned locally and hard copies forwarded to the coordinating centre. AHP questionnaire data entry will be completed by state-based research assistants using standardised spreadsheets; a minimum of 20% of questionnaire data will be double entered to determine data quality. Child data collected during the clinical assessment will be entered by the assessing AHP or state-based research assistant into the electronic cerebral palsy clinical outcomes database. This database will provide ‘real-time’ feedback to professionals about changes in children’s status on reassessment, flagging whether the child has lost function and requires immediate review or is progressing as expected. This process confers validity to both the measurement and data entry processes, as deviation from normal parameters is flagged in real time.

### Intellectual property

The evidence library (CP Decision) and electronic database are pre-existing intellectual property of the Cerebral Palsy Alliance and have been provided as an ‘in-kind’ contribution for use within this research project. Knowledge generated within this project will be the collective property of the research team. A publication plan will be developed by the steering committee, including identification of named authors based on contribution to each manuscript and acknowledgment of all team members and organisations.

### Dissemination

Study findings will be disseminated through publication in peer-reviewed journals and presentation at relevant academic and clinical conferences and forums. Key findings will also be available on the study web pages [40] and will be disseminated through partner organisations via presentations and circulation of summaries of findings. A lay summary will also be developed and circulated to participating children and families.

## Trial status

The study is ongoing. We have collected questionnaire data from AHPs and conducted focus groups but have not commenced data cleaning or analyses. We have not yet extracted data from the cerebral palsy clinical outcomes database. Data collection will continue until the end of 2015.

## Discussion

This project aims to reduce the research-practice gap in provision of AHP services to children with cerebral palsy via a multi-strategy intervention. The project has the potential to influence future practice with children with cerebral palsy. The demonstration of effective uptake of routine clinical assessment in multiple organisations will positively impact the implementation of outcome measurement in routine practice across Australia and internationally. The use of the electronic clinical outcomes database will support endeavours to link child outcomes data with diagnostic data held on the Australian Cerebral Palsy Register (ACPR). Together, these databases can enhance new knowledge about cerebral palsy.

With the implementation of the National Disability Insurance Scheme (NDIS) in Australia [32], service providers are seeking evidence-based information to support decision-making about how to distribute scarce resources, and families are seeking information to support intervention choices. This study will support the implementation of the NDIS with knowledge about what constitutes an efficient, evidence-informed service and which allied health interventions are currently implemented for children with cerebral palsy in Australia. Potential cost savings from this study include an ongoing saving in therapy dollars associated with ceasing the provision of the 20% of ineffective services to Australians with cerebral palsy [4-6], as a result of closing the research-practice gap. These cost savings could be redistributed to help children currently on waiting lists and used to provide effective therapies.

In the long term, this partnership project aims to embed systems, processes and professional behaviours within organisations in the health industry that will increase the responsiveness of health services to new evidence to inform clinical practice with children with cerebral palsy. The research utilisation model that includes identifying and supporting knowledge brokers, providing up-to-date summarised evidence via electronic platforms and routine data collection with real-time feedback will drive long-term, population-based research and practice. It will provide the structures and processes to successfully link research, AHP actions and child and family outcomes where the child has cerebral palsy.

## Abbreviations

AHP: Allied health professionals
NDIS: National disability insurance scheme
NHMRC: National Health and Medical Research Council
